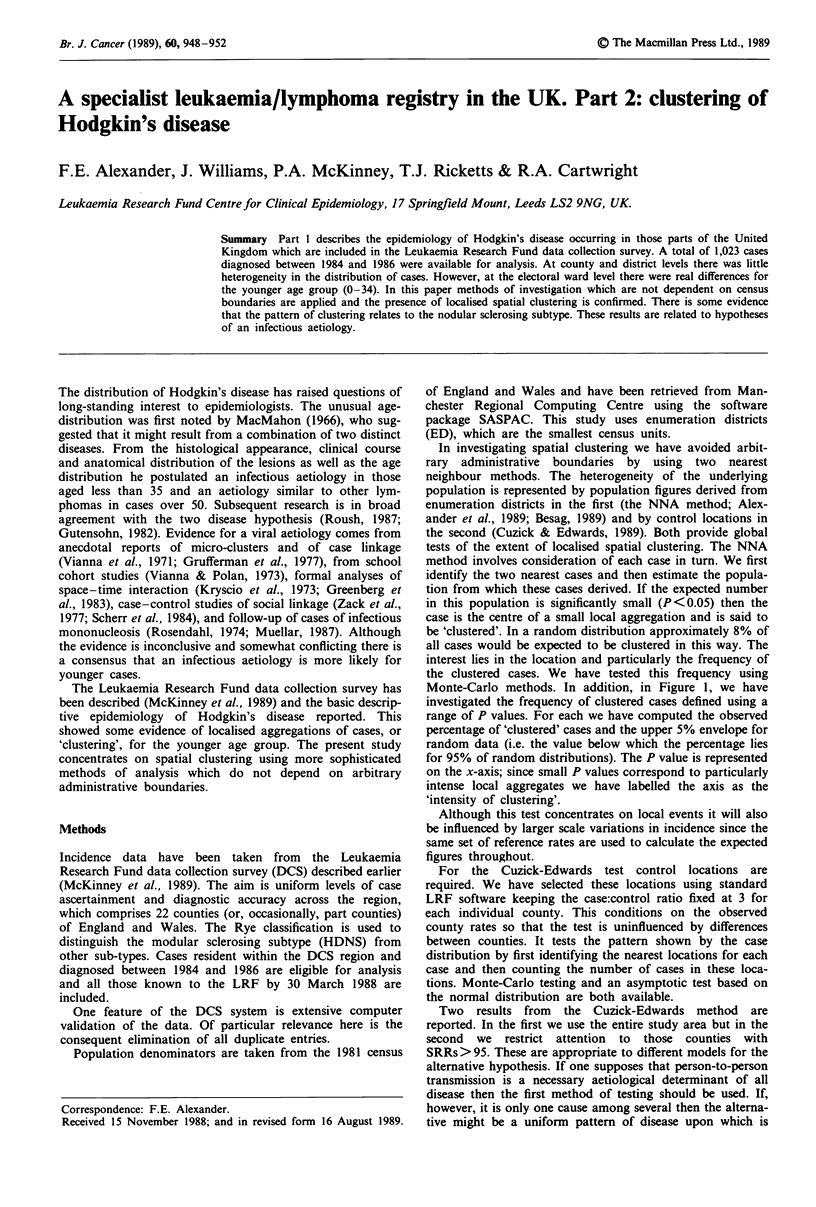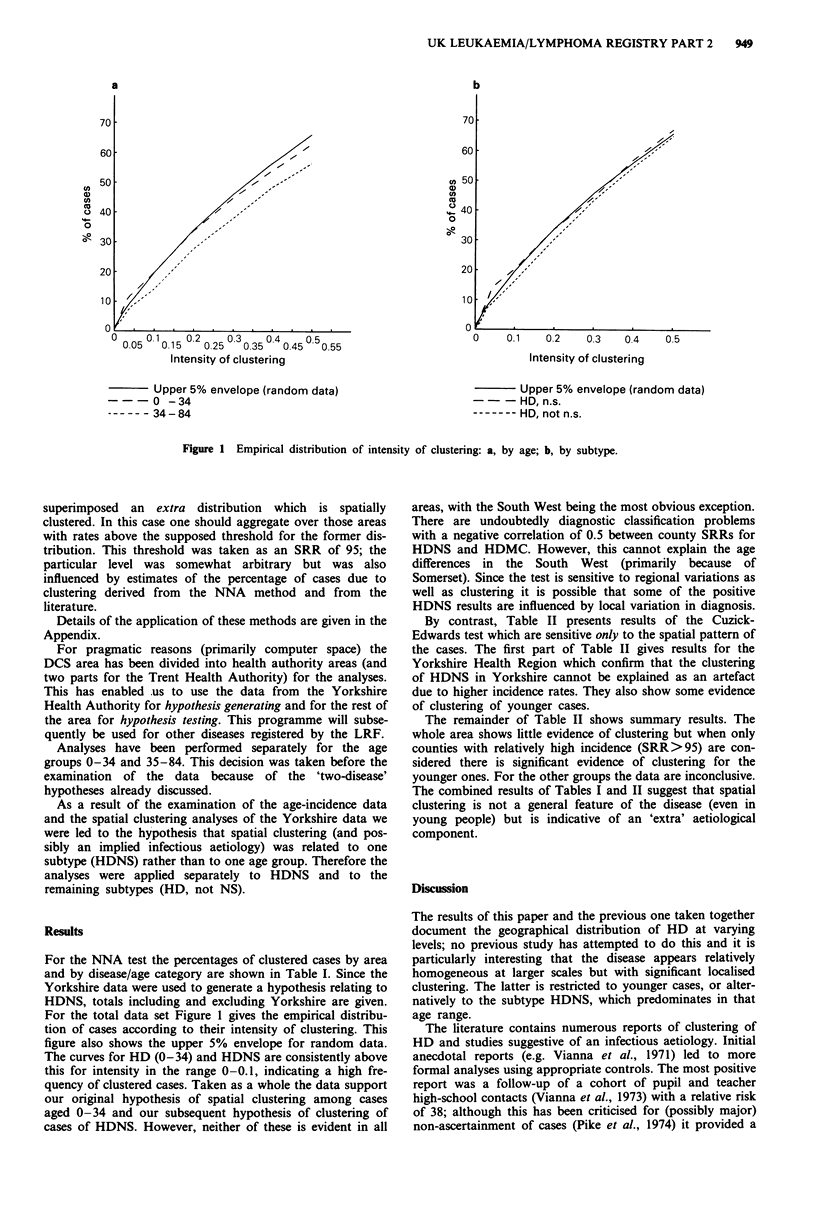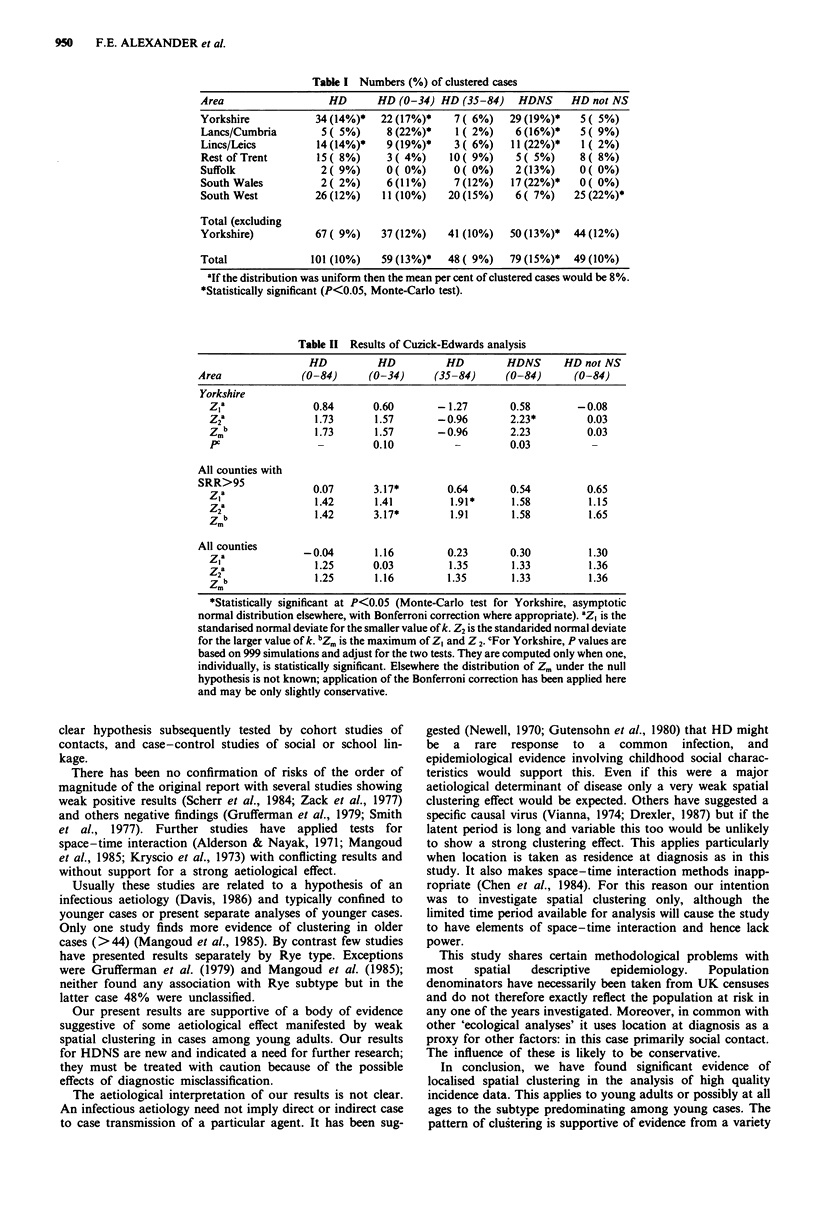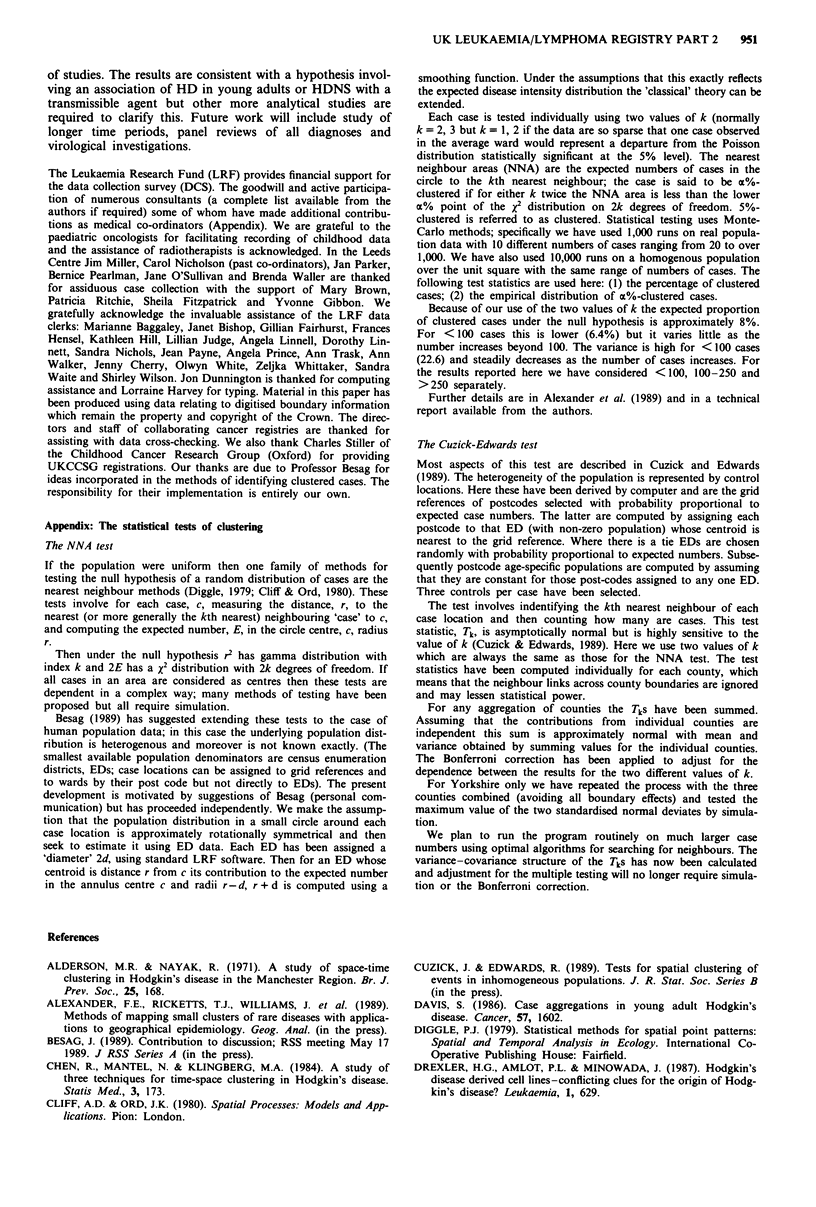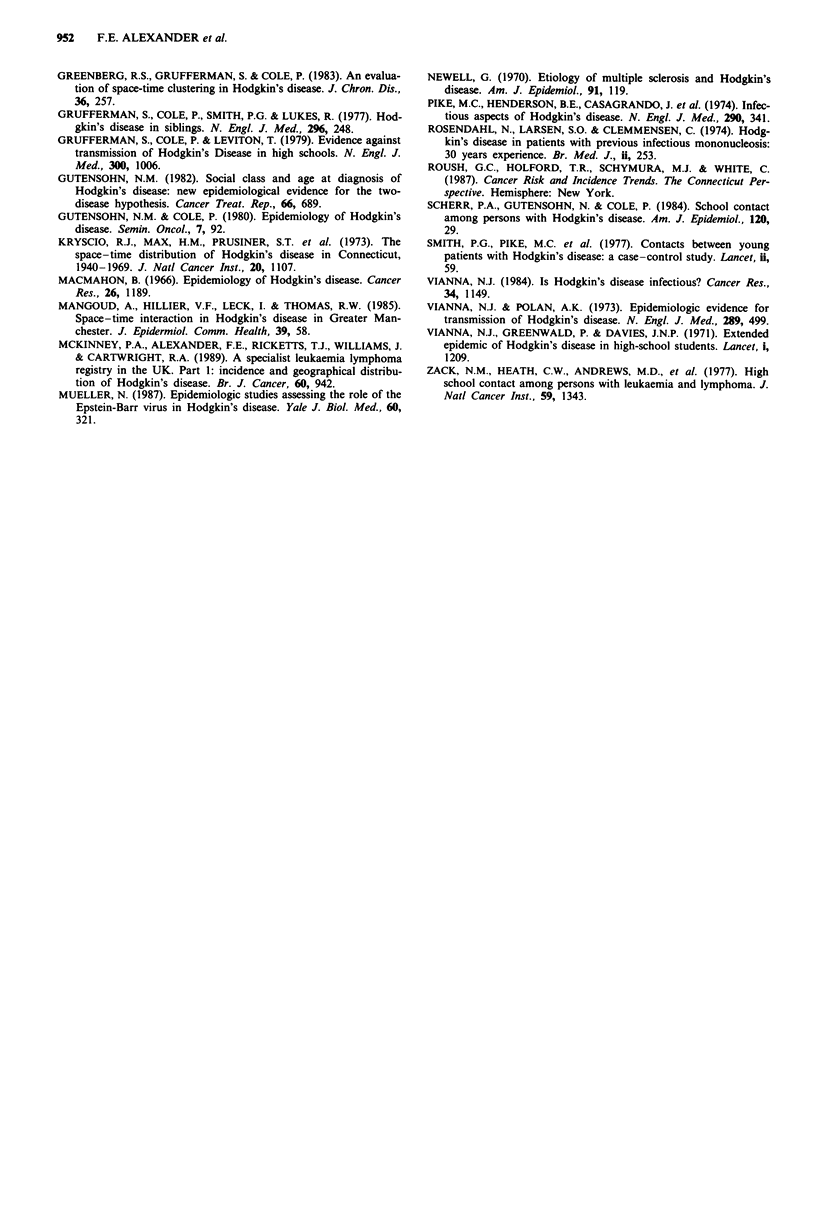# A specialist leukaemia/lymphoma registry in the UK. Part 2: Clustering of Hodgkin's disease.

**DOI:** 10.1038/bjc.1989.396

**Published:** 1989-12

**Authors:** F. E. Alexander, J. Williams, P. A. McKinney, T. J. Ricketts, R. A. Cartwright

**Affiliations:** Leukaemia Research Fund Centre for Clinical Epidemiology, Leeds, UK.

## Abstract

Part 1 describes the epidemiology of Hodgkin's disease occurring in those parts of the United Kingdom which are included in the Leukaemia Research Fund data collection survey. A total of 1,023 cases diagnosed between 1984 and 1986 were available for analysis. At county and district levels there was little heterogeneity in the distribution of cases. However, at the electoral ward level there were real differences for the younger age group (0-34). In this paper methods of investigation which are not dependent on census boundaries are applied and the presence of localised spatial clustering is confirmed. There is some evidence that the pattern of clustering relates to the nodular sclerosing subtype. These results are related to hypotheses of an infectious aetiology.


Br. J. Cancer (1989), 60, 948–952 © The Macmillan Press Ltd., 1989

### A specialist leukaemia/lymphoma registry in the UK. Part 2: clustering of Hodgkin's disease

F.E. Alexander, J. Williams, P.A. McKinney, T.J. Ricketts & R.A. Cartwright

*Leukaemia Research Fund Centre for Clinical Epidemiology, 17 Springfield Mount, Leeds LS2 9NG, UK.*

**Summary** Part 1 describes the epidemiology of Hodgkin's disease occurring in those parts of the United Kingdom which are included in the Leukaemia Research Fund data collection survey. A total of 1,023 cases diagnosed between 1984 and 1986 were available for analysis. At county and district levels there was little heterogeneity in the distribution of cases. However, at the electoral ward level there were real differences for the younger age group (0–34). In this paper methods of investigation which are not dependent on census boundaries are applied and the presence of localised spatial clustering is confirmed. There is some evidence that the pattern of clustering relates to the nodular sclerosing subtype. These results are related to hypotheses of an infectious aetiology.

The distribution of Hodgkin's disease has raised questions of long-standing interest to epidemiologists. The unusual age-distribution was first noted by MacMahon (1966), who suggested that it might result from a combination of two distinct diseases. From the histological appearance, clinical course and anatomical distribution of the lesions as well as the age distribution he postulated an infectious aetiology in those aged less than 35 and an aetiology similar to other lymphomas in cases over 50. Subsequent research is in broad agreement with the two disease hypothesis (Roush, 1987; Gutensohn, 1982). Evidence for a viral aetiology comes from anecdotal reports of micro-clusters and of case linkage (Vianna *et al.*, 1971; Grufferman *et al.*, 1977), from school cohort studies (Vianna & Polan, 1973), formal analyses of space–time interaction (Kryscio *et al.*, 1973; Greenberg *et al.*, 1983), case–control studies of social linkage (Zack *et al.*, 1977; Scherr *et al.*, 1984), and follow-up of cases of infectious mononucleosis (Rosendahl, 1974; Muellar, 1987). Although the evidence is inconclusive and somewhat conflicting there is a consensus that an infectious aetiology is more likely for younger cases.

The Leukaemia Research Fund data collection survey has been described (McKinney *et al.*, 1989) and the basic descriptive epidemiology of Hodgkin's disease reported. This showed some evidence of localised aggregations of cases, or 'clustering', for the younger age group. The present study concentrates on spatial clustering using more sophisticated methods of analysis which do not depend on arbitrary administrative boundaries.

### Methods

Incidence data have been taken from the Leukaemia Research Fund data collection survey (DCS) described earlier (McKinney *et al.*, 1989). The aim is uniform levels of case ascertainment and diagnostic accuracy across the region, which comprises 22 counties (or, occasionally, part counties) of England and Wales. The Rye classification is used to distinguish the modular sclerosing subtype (HDNS) from other sub-types. Cases resident within the DCS region and diagnosed between 1984 and 1986 are eligible for analysis and all those known to the LRF by 30 March 1988 are included.

One feature of the DCS system is extensive computer validation of the data. Of particular relevance here is the consequent elimination of all duplicate entries.

Population denominators are taken from the 1981 census of England and Wales and have been retrieved from Manchester Regional Computing Centre using the software package SASPAC. This study uses enumeration districts (ED), which are the smallest census units.

In investigating spatial clustering we have avoided arbitrary administrative boundaries by using two nearest neighbour methods. The heterogeneity of the underlying population is represented by population figures derived from enumeration districts in the first (the NNA method; Alexander *et al.*, 1989; Besag, 1989) and by control locations in the second (Cuzick & Edwards, 1989). Both provide global tests of the extent of localised spatial clustering. The NNA method involves consideration of each case in turn. We first identify the two nearest cases and then estimate the population from which these cases derived. If the expected number in this population is significantly small ($P < 0.05$) then the case is the centre of a small local aggregation and is said to be 'clustered'. In a random distribution approximately 8% of all cases would be expected to be clustered in this way. The interest lies in the location and particularly the frequency of the clustered cases. We have tested this frequency using Monte-Carlo methods. In addition, in Figure 1, we have investigated the frequency of clustered cases defined using a range of $P$ values. For each we have computed the observed percentage of 'clustered' cases and the upper 5% envelope for random data (i.e. the value below which the percentage lies for 95% of random distributions). The $P$ value is represented on the $x$-axis; since small $P$ values correspond to particularly intense local aggregates we have labelled the axis as the 'intensity of clustering'.

Although this test concentrates on local events it will also be influenced by larger scale variations in incidence since the same set of reference rates are used to calculate the expected figures throughout.

For the Cuzick-Edwards test control locations are required. We have selected these locations using standard LRF software keeping the case:control ratio fixed at 3 for each individual county. This conditions on the observed county rates so that the test is uninfluenced by differences between counties. It tests the pattern shown by the case distribution by first identifying the nearest locations for each case and then counting the number of cases in these locations. Monte-Carlo testing and an asymptotic test based on the normal distribution are both available.

Two results from the Cuzick-Edwards method are reported. In the first we use the entire study area but in the second we restrict attention to those counties with SRRs > 95. These are appropriate to different models for the alternative hypothesis. If one supposes that person-to-person transmission is a necessary aetiological determinant of all disease then the first method of testing should be used. If, however, it is only one cause among several then the alternative might be a uniform pattern of disease upon which is

Correspondence: F.E. Alexander.
Received 15 November 1988; and in revised form 16 August 1989.

**Figure 1** Empirical distribution of intensity of clustering: **a**, by age; **b**, by subtype.

superimposed an *extra* distribution which is spatially clustered. In this case one should aggregate over those areas with rates above the supposed threshold for the former distribution. This threshold was taken as an SRR of 95; the particular level was somewhat arbitrary but was also influenced by estimates of the percentage of cases due to clustering derived from the NNA method and from the literature.

Details of the application of these methods are given in the Appendix.

For pragmatic reasons (primarily computer space) the DCS area has been divided into health authority areas (and two parts for the Trent Health Authority) for the analyses. This has enabled us to use the data from the Yorkshire Health Authority for *hypothesis generating* and for the rest of the area for *hypothesis testing*. This programme will subsequently be used for other diseases registered by the LRF.

Analyses have been performed separately for the age groups 0–34 and 35–84. This decision was taken before the examination of the data because of the 'two-disease' hypotheses already discussed.

As a result of the examination of the age-incidence data and the spatial clustering analyses of the Yorkshire data we were led to the hypothesis that spatial clustering (and possibly an implied infectious aetiology) was related to one subtype (HDNS) rather than to one age group. Therefore the analyses were applied separately to HDNS and to the remaining subtypes (HD, not NS).

### Results

For the NNA test the percentages of clustered cases by area and by disease/age category are shown in Table I. Since the Yorkshire data were used to generate a hypothesis relating to HDNS, totals including and excluding Yorkshire are given. For the total data set Figure 1 gives the empirical distribution of cases according to their intensity of clustering. This figure also shows the upper 5% envelope for random data. The curves for HD (0–34) and HDNS are consistently above this for intensity in the range 0–0.1, indicating a high frequency of clustered cases. Taken as a whole the data support our original hypothesis of spatial clustering among cases aged 0–34 and our subsequent hypothesis of clustering of cases of HDNS. However, neither of these is evident in all areas, with the South West being the most obvious exception. There are undoubtedly diagnostic classification problems with a negative correlation of 0.5 between county SRRs for HDNS and HDMC. However, this cannot explain the age differences in the South West (primarily because of Somerset). Since the test is sensitive to regional variations as well as clustering it is possible that some of the positive HDNS results are influenced by local variation in diagnosis.

By contrast, Table II presents results of the Cuzick-Edwards test which are sensitive *only* to the spatial pattern of the cases. The first part of Table II gives results for the Yorkshire Health Region which confirm that the clustering of HDNS in Yorkshire cannot be explained as an artefact due to higher incidence rates. They also show some evidence of clustering of younger cases.

The remainder of Table II shows summary results. The whole area shows little evidence of clustering but when only counties with relatively high incidence (SRR>95) are considered there is significant evidence of clustering for the younger ones. For the other groups the data are inconclusive. The combined results of Tables I and II suggest that spatial clustering is not a general feature of the disease (even in young people) but is indicative of an 'extra' aetiological component.

### Discussion

The results of this paper and the previous one taken together document the geographical distribution of HD at varying levels; no previous study has attempted to do this and it is particularly interesting that the disease appears relatively homogeneous at larger scales but with significant localised clustering. The latter is restricted to younger cases, or alternatively to the subtype HDNS, which predominates in that age range.

The literature contains numerous reports of clustering of HD and studies suggestive of an infectious aetiology. Initial anecdotal reports (e.g. Vianna *et al.*, 1971) led to more formal analyses using appropriate controls. The most positive report was a follow-up of a cohort of pupil and teacher high-school contacts (Vianna *et al.*, 1973) with a relative risk of 38; although this has been criticised for (possibly major) non-ascertainment of cases (Pike *et al.*, 1974) it provided a

**Table I** Numbers (%) of clustered cases

| *Area* | *HD* | *HD (0–34)* | *HD (35–84)* | *HDNS* | *HD not NS* |
|---|---|---|---|---|---|
| Yorkshire | 34 (14%)* | 22 (17%)* | 7 ( 6%) | 29 (19%)* | 5 ( 5%) |
| Lancs/Cumbria | 5 ( 5%) | 8 (22%)* | 1 ( 2%) | 6 (16%)* | 5 ( 9%) |
| Lincs/Leics | 14 (14%)* | 9 (19%)* | 3 ( 6%) | 11 (22%)* | 1 ( 2%) |
| Rest of Trent | 15 ( 8%) | 3 ( 4%) | 10 ( 9%) | 5 ( 5%) | 8 ( 8%) |
| Suffolk | 2 ( 9%) | 0 ( 0%) | 0 ( 0%) | 2 (13%) | 0 ( 0%) |
| South Wales | 2 ( 2%) | 6 (11%) | 7 (12%) | 17 (22%)* | 0 ( 0%) |
| South West | 26 (12%) | 11 (10%) | 20 (15%) | 6 ( 7%) | 25 (22%)* |
| Total (excluding Yorkshire) | 67 ( 9%) | 37 (12%) | 41 (10%) | 50 (13%)* | 44 (12%) |
| Total | 101 (10%) | 59 (13%)* | 48 ( 9%) | 79 (15%)* | 49 (10%) |

[a]If the distribution was uniform then the mean per cent of clustered cases would be 8%.
*Statistically significant ($P<0.05$, Monte-Carlo test).

**Table II** Results of Cuzick-Edwards analysis

| *Area* | *HD (0–84)* | *HD (0–34)* | *HD (35–84)* | *HDNS (0–84)* | *HD not NS (0–84)* |
|---|---|---|---|---|---|
| *Yorkshire* | | | | | |
| $Z_1$[a] | 0.84 | 0.60 | −1.27 | 0.58 | −0.08 |
| $Z_2$[a] | 1.73 | 1.57 | −0.96 | 2.23* | 0.03 |
| $Z_m$[b] | 1.73 | 1.57 | −0.96 | 2.23 | 0.03 |
| $P$[c] | – | 0.10 | – | 0.03 | – |
| All counties with SRR>95 | | | | | |
| $Z_1$[a] | 0.07 | 3.17* | 0.64 | 0.54 | 0.65 |
| $Z_2$[a] | 1.42 | 1.41 | 1.91* | 1.58 | 1.15 |
| $Z_m$[b] | 1.42 | 3.17* | 1.91 | 1.58 | 1.65 |
| All counties | | | | | |
| $Z_1$[a] | −0.04 | 1.16 | 0.23 | 0.30 | 1.30 |
| $Z_2$[a] | 1.25 | 0.03 | 1.35 | 1.33 | 1.36 |
| $Z_m$[b] | 1.25 | 1.16 | 1.35 | 1.33 | 1.36 |

*Statistically significant at $P<0.05$ (Monte-Carlo test for Yorkshire, asymptotic normal distribution elsewhere, with Bonferroni correction where appropriate). [a]$Z_1$ is the standarised normal deviate for the smaller value of $k$. $Z_2$ is the standarided normal deviate for the larger value of $k$. [b]$Z_m$ is the maximum of $Z_1$ and $Z_2$. [c]For Yorkshire, $P$ values are based on 999 simulations and adjust for the two tests. They are computed only when one, individually, is statistically significant. Elsewhere the distribution of $Z_m$ under the null hypothesis is not known; application of the Bonferroni correction has been applied here and may be only slightly conservative.

clear hypothesis subsequently tested by cohort studies of contacts, and case–control studies of social or school linkage.

There has been no confirmation of risks of the order of magnitude of the original report with several studies showing weak positive results (Scherr *et al.*, 1984; Zack *et al.*, 1977) and others negative findings (Grufferman *et al.*, 1979; Smith *et al.*, 1977). Further studies have applied tests for space–time interaction (Alderson & Nayak, 1971; Mangoud *et al.*, 1985; Kryscio *et al.*, 1973) with conflicting results and without support for a strong aetiological effect.

Usually these studies are related to a hypothesis of an infectious aetiology (Davis, 1986) and typically confined to younger cases or present separate analyses of younger cases. Only one study finds more evidence of clustering in older cases (>44) (Mangoud *et al.*, 1985). By contrast few studies have presented results separately by Rye type. Exceptions were Grufferman *et al.* (1979) and Mangoud *et al.* (1985); neither found any association with Rye subtype but in the latter case 48% were unclassified.

Our present results are supportive of a body of evidence suggestive of some aetiological effect manifested by weak spatial clustering in cases among young adults. Our results for HDNS are new and indicated a need for further research; they must be treated with caution because of the possible effects of diagnostic misclassification.

The aetiological interpretation of our results is not clear. An infectious aetiology need not imply direct or indirect case to case transmission of a particular agent. It has been suggested (Newell, 1970; Gutensohn *et al.*, 1980) that HD might be a rare response to a common infection, and epidemiological evidence involving childhood social characteristics would support this. Even if this were a major aetiological determinant of disease only a very weak spatial clustering effect would be expected. Others have suggested a specific causal virus (Vianna, 1974; Drexler, 1987) but if the latent period is long and variable this too would be unlikely to show a strong clustering effect. This applies particularly when location is taken as residence at diagnosis as in this study. It also makes space–time interaction methods inappropriate (Chen *et al.*, 1984). For this reason our intention was to investigate spatial clustering only, although the limited time period available for analysis will cause the study to have elements of space–time interaction and hence lack power.

This study shares certain methodological problems with most spatial descriptive epidemiology. Population denominators have necessarily been taken from UK censuses and do not therefore exactly reflect the population at risk in any one of the years investigated. Moreover, in common with other 'ecological analyses' it uses location at diagnosis as a proxy for other factors: in this case primarily social contact. The influence of these is likely to be conservative.

In conclusion, we have found significant evidence of localised spatial clustering in the analysis of high quality incidence data. This applies to young adults or possibly at all ages to the subtype predominating among young cases. The pattern of clustering is supportive of evidence from a variety of studies. The results are consistent with a hypothesis involving an association of HD in young adults or HDNS with a transmissible agent but other more analytical studies are required to clarify this. Future work will include study of longer time periods, panel reviews of all diagnoses and virological investigations.

The Leukaemia Research Fund (LRF) provides financial support for the data collection survey (DCS). The goodwill and active participation of numerous consultants (a complete list available from the authors if required) some of whom have made additional contributions as medical co-ordinators (Appendix). We are grateful to the paediatric oncologists for facilitating recording of childhood data and the assistance of radiotherapists is acknowledged. In the Leeds Centre Jim Miller, Carol Nicholson (past co-ordinators), Jan Parker, Bernice Pearlman, Jane O'Sullivan and Brenda Waller are thanked for assiduous case collection with the support of Mary Brown, Patricia Ritchie, Sheila Fitzpatrick and Yvonne Gibbon. We gratefully acknowledge the invaluable assistance of the LRF data clerks: Marianne Baggaley, Janet Bishop, Gillian Fairhurst, Frances Hensel, Kathleen Hill, Lillian Judge, Angela Linnell, Dorothy Linnett, Sandra Nichols, Jean Payne, Angela Prince, Ann Trask, Ann Walker, Jenny Cherry, Olwyn White, Zeljka Whittaker, Sandra Waite and Shirley Wilson. Jon Dunnington is thanked for computing assistance and Lorraine Harvey for typing. Material in this paper has been produced using data relating to digitised boundary information which remain the property and copyright of the Crown. The directors and staff of collaborating cancer registries are thanked for assisting with data cross-checking. We also thank Charles Stiller of the Childhood Cancer Research Group (Oxford) for providing UKCCSG registrations. Our thanks are due to Professor Besag for ideas incorporated in the methods of identifying clustered cases. The responsibility for their implementation is entirely our own.

### Appendix: The statistical tests of clustering

### *The NNA test*

If the population were uniform then one family of methods for testing the null hypothesis of a random distribution of cases are the nearest neighbour methods (Diggle, 1979; Cliff & Ord, 1980). These tests involve for each case, $c$, measuring the distance, $r$, to the nearest (or more generally the $k$th nearest) neighbouring 'case' to $c$, and computing the expected number, $E$, in the circle centre, $c$, radius $r$.

Then under the null hypothesis $r^2$ has gamma distribution with index $k$ and $2E$ has a $\chi^2$ distribution with $2k$ degrees of freedom. If all cases in an area are considered as centres then these tests are dependent in a complex way; many methods of testing have been proposed but all require simulation.

Besag (1989) has suggested extending these tests to the case of human population data; in this case the underlying population distribution is heterogenous and moreover is not known exactly. (The smallest available population denominators are census enumeration districts, EDs; case locations can be assigned to grid references and to wards by their post code but not directly to EDs). The present development is motivated by suggestions of Besag (personal communication) but has proceeded independently. We make the assumption that the population distribution in a small circle around each case location is approximately rotationally symmetrical and then seek to estimate it using ED data. Each ED has been assigned a 'diameter' $2d$, using standard LRF software. Then for an ED whose centroid is distance $r$ from $c$ its contribution to the expected number in the annulus centre $c$ and radii $r-d$, $r+\mathrm{d}$ is computed using a smoothing function. Under the assumptions that this exactly reflects the expected disease intensity distribution the 'classical' theory can be extended.

Each case is tested individually using two values of $k$ (normally $k=2, 3$ but $k=1, 2$ if the data are so sparse that one case observed in the average ward would represent a departure from the Poisson distribution statistically significant at the 5% level). The nearest neighbour areas (NNA) are the expected numbers of cases in the circle to the $k$th nearest neighbour; the case is said to be $\alpha$%-clustered if for either $k$ twice the NNA area is less than the lower $\alpha$% point of the $\chi^2$ distribution on $2k$ degrees of freedom. 5%-clustered is referred to as clustered. Statistical testing uses Monte-Carlo methods; specifically we have used 1,000 runs on real population data with 10 different numbers of cases ranging from 20 to over 1,000. We have also used 10,000 runs on a homogenous population over the unit square with the same range of numbers of cases. The following test statistics are used here: (1) the percentage of clustered cases; (2) the empirical distribution of $\alpha$%-clustered cases.

Because of our use of the two values of $k$ the expected proportion of clustered cases under the null hypothesis is approximately 8%. For $<100$ cases this is lower (6.4%) but it varies little as the number increases beyond 100. The variance is high for $<100$ cases (22.6) and steadily decreases as the number of cases increases. For the results reported here we have considered $<100$, 100–250 and $>250$ separately.

Further details are in Alexander *et al.* (1989) and in a technical report available from the authors.

### *The Cuzick-Edwards test*

Most aspects of this test are described in Cuzick and Edwards (1989). The heterogeneity of the population is represented by control locations. Here these have been derived by computer and are the grid references of postcodes selected with probability proportional to expected case numbers. The latter are computed by assigning each postcode to that ED (with non-zero population) whose centroid is nearest to the grid reference. Where there is a tie EDs are chosen randomly with probability proportional to expected numbers. Subsequently postcode age-specific populations are computed by assuming that they are constant for those post-codes assigned to any one ED. Three controls per case have been selected.

The test involves indentifying the $k$th nearest neighbour of each case location and then counting how many are cases. This test statistic, $T_k$, is asymptotically normal but is highly sensitive to the value of $k$ (Cuzick & Edwards, 1989). Here we use two values of $k$ which are always the same as those for the NNA test. The test statistics have been computed individually for each county, which means that the neighbour links across county boundaries are ignored and may lessen statistical power.

For any aggregation of counties the $T_k$s have been summed. Assuming that the contributions from individual counties are independent this sum is approximately normal with mean and variance obtained by summing values for the individual counties. The Bonferroni correction has been applied to adjust for the dependence between the results for the two different values of $k$.

For Yorkshire only we have repeated the process with the three counties combined (avoiding all boundary effects) and tested the maximum value of the two standardised normal deviates by simulation.

We plan to run the program routinely on much larger case numbers using optimal algorithms for searching for neighbours. The variance–covariance structure of the $T_k$s has now been calculated and adjustment for the multiple testing will no longer require simulation or the Bonferroni correction.

### References

ALDERSON, M.R. & NAYAK, R. (1971). A study of space-time clustering in Hodgkin's disease in the Manchester Region. *Br. J. Prev. Soc.*, **25**, 168.

ALEXANDER, F.E., RICKETTS, T.J., WILLIAMS, J. *et al.* (1989). Methods of mapping small clusters of rare diseases with applications to geographical epidemiology. *Geog. Anal.* (in the press).

BESAG, J. (1989). Contribution to discussion; RSS meeting May 17 1989. *J RSS Series A* (in the press).

CHEN, R., MANTEL, N. & KLINGBERG, M.A. (1984). A study of three techniques for time-space clustering in Hodgkin's disease. *Statis Med.*, **3**, 173.

CLIFF, A.D. & ORD, J.K. (1980). *Spatial Processes: Models and Applications.* Pion: London.

CUZICK, J. & EDWARDS, R. (1989). Tests for spatial clustering of events in inhomogeneous populations. *J. R. Stat. Soc. Series B* (in the press).

DAVIS, S. (1986). Case aggregations in young adult Hodgkin's disease. *Cancer*, **57**, 1602.

DIGGLE, P.J. (1979). Statistical methods for spatial point patterns: *Spatial and Temporal Analysis in Ecology.* International Co-Operative Publishing House: Fairfield.

DREXLER, H.G., AMLOT, P.L. & MINOWADA, J. (1987). Hodgkin's disease derived cell lines–conflicting clues for the origin of Hodgkin's disease? *Leukaemia*, **1**, 629.

GREENBERG, R.S., GRUFFERMAN, S. & COLE, P. (1983). An evaluation of space-time clustering in Hodgkin's disease. *J. Chron. Dis.*, **36**, 257.

GRUFFERMAN, S., COLE, P., SMITH, P.G. & LUKES, R. (1977). Hodgkin's disease in siblings. *N. Engl. J. Med.*, **296**, 248.

GRUFFERMAN, S., COLE, P. & LEVITON, T. (1979). Evidence against transmission of Hodgkin's Disease in high schools. *N. Engl. J. Med.*, **300**, 1006.

GUTENSOHN, N.M. (1982). Social class and age at diagnosis of Hodgkin's disease: new epidemiological evidence for the two-disease hypothesis. *Cancer Treat. Rep.*, **66**, 689.

GUTENSOHN, N.M. & COLE, P. (1980). Epidemiology of Hodgkin's disease. *Semin. Oncol.*, **7**, 92.

KRYSCIO, R.J., MAX, H.M., PRUSINER, S.T. *et al.* (1973). The space–time distribution of Hodgkin's disease in Connecticut, 1940–1969. *J. Natl Cancer Inst.*, **20**, 1107.

MACMAHON, B. (1966). Epidemiology of Hodgkin's disease. *Cancer Res.*, **26**, 1189.

MANGOUD, A., HILLIER, V.F., LECK, I. & THOMAS, R.W. (1985). Space–time interaction in Hodgkin's disease in Greater Manchester. *J. Epidermiol. Comm. Health*, **39**, 58.

MCKINNEY, P.A., ALEXANDER, F.E., RICKETTS, T.J., WILLIAMS, J. & CARTWRIGHT, R.A. (1989). A specialist leukaemia lymphoma registry in the UK. Part 1: incidence and geographical distribution of Hodgkin's disease. *Br. J. Cancer*, **60**, 942.

MUELLER, N. (1987). Epidemiologic studies assessing the role of the Epstein-Barr virus in Hodgkin's disease. *Yale J. Biol. Med.*, **60**, 321.

NEWELL, G. (1970). Etiology of multiple sclerosis and Hodgkin's disease. *Am. J. Epidemiol.*, **91**, 119.

PIKE, M.C., HENDERSON, B.E., CASAGRANDO, J. *et al.* (1974). Infectious aspects of Hodgkin's disease. *N. Engl. J. Med.*, **290**, 341.

ROSENDAHL, N., LARSEN, S.O. & CLEMMENSEN, C. (1974). Hodgkin's disease in patients with previous infectious mononucleosis: 30 years experience. *Br. Med. J.*, **ii**, 253.

ROUSH, G.C., HOLFORD, T.R., SCHYMURA, M.J. & WHITE, C. (1987). *Cancer Risk and Incidence Trends. The Connecticut Perspective*. Hemisphere: New York.

SCHERR, P.A., GUTENSOHN, N. & COLE, P. (1984). School contact among persons with Hodgkin's disease. *Am. J. Epidemiol.*, **120**, 29.

SMITH, P.G., PIKE, M.C. *et al.* (1977). Contacts between young patients with Hodgkin's disease: a case–control study. *Lancet*, **ii**, 59.

VIANNA, N.J. (1984). Is Hodgkin's disease infectious? *Cancer Res.*, **34**, 1149.

VIANNA, N.J. & POLAN, A.K. (1973). Epidemiologic evidence for transmission of Hodgkin's disease. *N. Engl. J. Med.*, **289**, 499.

VIANNA, N.J., GREENWALD, P. & DAVIES, J.N.P. (1971). Extended epidemic of Hodgkin's disease in high-school students. *Lancet*, **i**, 1209.

ZACK, N.M., HEATH, C.W., ANDREWS, M.D., *et al.* (1977). High school contact among persons with leukaemia and lymphoma. *J. Natl Cancer Inst.*, **59**, 1343.